# Preliminary study for predicting better methotrexate efficacy in Japanese patients with rheumatoid arthritis

**DOI:** 10.1186/s40780-016-0047-6

**Published:** 2016-06-07

**Authors:** Masayuki Hashiguchi, Tomomi Tsuru, Kumika Miyawaki, Midori Suzaki, Jun Hakamata, Mikiko Shimizu, Shin Irie, Mayumi Mochizuki

**Affiliations:** Division for Evaluation and Analysis of Drug Information, Faculty of Pharmacy, Keio University, 1-5-30 Shibakoen, Minato-ku, Tokyo, 105-8512 Japan; PS Clinic, LTA Clinical Pharmacology Center, 6-18 Tenyacho, Hakata-ku, Fukuoka, 812-0025 Japan; Department of Hygienic Chemistry, Faculty of Pharmacy, Keio University, 1-5-30 Shibakoen, Minato-ku, Tokyo, 105-8512 Japan; LTA Clinical Pharmacology Center, 6-18 Tenyacho, Hakata-ku, Fukuoka, 812-0025 Japan

**Keywords:** Methotrexate, Rheumatoid arthritis, Therapeutic response, Interpatient variability, DAS28, Genetic polymorphism

## Abstract

**Background:**

Rheumatoid arthritis (RA) is a chronic autoimmune disease characterized by systemic inflammatory status, joint destruction, disability, and pain. Methotrexate (MTX) has been confirmed to reduce disease activity and delay or stabilize the development of bone erosions. However, major drawbacks are that patients show great interindividual variability in response to MTX and the unpredictable occurrence of side effects. A strategy for personalized MTX treatment to predict its efficacy and toxicity has not yet been determined.

To establish personalized MTX therapy in Japanese patients with rheumatoid arthritis, we performed a preliminary study for predicting better methotrexate efficacy including single-nucleotide polymorphisms (SNPs) for MTX-related transporters/enzymes.

**Methods:**

Disease control status (good or poor) was judged by the number of Disease Activity Scores (DAS28) of <2 for 6–12 months. The response index R was calculated by the improved area under the curve (AUC) of the DAS28 score for 0–3 or 0–6 months by dividing the cumulative dose of MTX during 0–3 or 0–6 months, respectively. Genotyping of alleles of RFC1 80G > A, RFC1 –43 T > C, FPGS 1994G > A, GGH 401C > T, MTHFR 1298A > C, and TYMS 3'-UTR (−6/+6) was performed using the real-time PCR system.

**Results:**

Seven of 21 patients were judged as good responders in terms of disease control, and the remainder as poor responders. For 0–3 months after starting MTX administration, the median cumulative dose and improved DAS28 AUC in the good and poor response groups were 96.0 mg and 25.4 and 118.0 mg and 23.4, respectively. For 0–6 months, the median cumulative dose and improved DAS28 AUC in the good and poor response groups were 192.0 mg and 51.0 and 214.0 mg and 47.6, respectively. Statistically significant differences between the 2 groups in the 0–6-month period were observed in DAS28 AUC improvement and index R. A slight tendency for a correlation between G/G genotypes and A allele genotypes in RFC1 80 genotypes was observed, although it did not reach statistical significance.

**Conclusion:**

This study suggested that aggressive RA treatment with MTX from the early period of administration is necessary to obtain a good response after 6 months, although no SNPs predicting a better treatment response to MTX were identified.

## Background

Rheumatoid arthritis (RA) is a chronic autoimmune disease characterized by systemic inflammatory status, joint destruction, disability, and pain [1, 2]. However, the mechanism of RA onset is not fully clear. Currently, clinical remission, that is, complete suppression of disease activity, is considered the major goal of RA treatment [3–6], and a significant proportion of patients receiving routine follow-up care can achieve this [7–9], although long-term drug therapy is required. Among agents for the treatment of RA, methotrexate (MTX) is the anchor drug and the most widely used disease-modifying antirheumatoid drug (DMARD). MTX has been confirmed to reduce disease activity and delay or stabilize the development of bone erosions [10, 11]. However, major drawbacks are that patients show great interindividual variability in response to MTX, only about 50 % show a good clinical response, and the unpredictable occurrence of a broad spectrum of side effects including alopecia, gastrointestinal disturbances, elevation of liver enzyme levels, and bone marrow suppression [12, 13] forces 30 % of patients to discontinue therapy [14, 15].

In the body, MTX is transported intracellularly via the reduced folate carrier 1 (RFC-1/SLC19A1). Inside cells [16, 17], MTX adds up to 4 additional glutamate moieties via folypolyglutamate synthetase (FPGS) [18–20] and then forms MTX-polyglutamates (MTXPGs). Subsequently, the terminal glutamate MTXPG molecules are removed via gamma-glutamyl hydrolase (GGH) [21–23] and returned to MTX (which is the MTX monoglutamate form) and it is rapidly transported out of the cell by multidrug-resistant proteins. The mechanism of action of MTX is as a folate antagonist [24], and therefore intracellular MTXPGs bind to dihydrofolate reductase (DHFR) and other folate pathway enzymes, thereby exerting antiinflammatory effects, although the detailed mechanism of action remains unclear.

Despite many efforts to identify factors predicting the response to MTX treatment, which have focused on drug disposition including single-nucleotide polymorphisms (SNPs) in genes coding for folate pathway enzymes and MTX transport into and out of cells in patients with RA in relation to MTX efficacy and toxicity [25–32], a strategy for personalized MTX treatment to predict its efficacy and toxicity has not yet been determined.

The purpose of this study was to clarify the relationship between treatment response to MTX and disease control status, identify the genetic polymorphisms for MTX-related transporters/enzymes, and obtain better supportive information to devise a strategy for MTX administration for personalized therapy in Japanese patients with RA.

## Methods

### Participants, study design, and setting

This was a retrospective observational cohort study. The patients included were unrelated Japanese with RA who regularly visited the PS Clinic (Fukuoka, Japan) from December 2010 to January 2011 and had been administered MTX continuously for 6 months or more, and/or were administered MTX for the first time. All patients gave written informed consent for participation in this study after receiving an explanation of the protocol and goals, especially in order to determine the SNPs related to the MTX treatment response from blood samples. The patients’ clinical data were retrospectively collected from their medical records. Patients who were receiving the combination of low-dose corticosteroids and nonsteroidal antiinflammatory drugs at the same fixed dose during the study period were included. Patients who received biologic agents were excluded from the study.

The study protocol was approved by the Ethics Committee of the LTA Clinical Pharmacology Center and Faculty of Pharmacy, Keio University.

### Study drug

The proprietary MTX brands used were Rheumatrex 2-mg capsules (Pfizer, Inc., Tokyo, Japan), Metolate 2-mg tablets (Santen Pharmaceutical Co., Ltd., Osaka, Japan), and Methotrexate 2-mg tablets (Mitsubishi Tanabe Pharma, Tokyo, Japan). The dose of MTX was determined by individual patients’ physicians based on stable clinical symptoms. The MTX doses when discontinuation was required due to the appearance of drug-related side effects were excluded from the study analysis.

### Patient characteristics

Gender, age at the commencement of MTX administration, C-reactive protein (CRP) level, erythrocyte sedimentation rate (ESR), rheumatoid factor (RF) level, Disease Activity Score 28-CRP (DAS28-CRP), serum creatinine (Scr), duration of RA, and history of DMARD administration were taken from patients’ medical records.

### Outcome measures

#### Evaluation of treatment response to MTX and disease control

DAS28-CRP was used as the index of disease activity status. DAS28 was calculated from the following formula with the number of tender joints from among 28 joints (T28), number of swollen joints (S28), Visual Analogue Scale (VAS) score, and CRP level [33].

$$ DAS28 = 0.56 \times \sqrt{T28} + 0.28 \times \sqrt{S28} + 0.36\  ln\left(CRP + 1\right) + 0.014 \times VAS + 0.96 $$

**Fig. 1:**
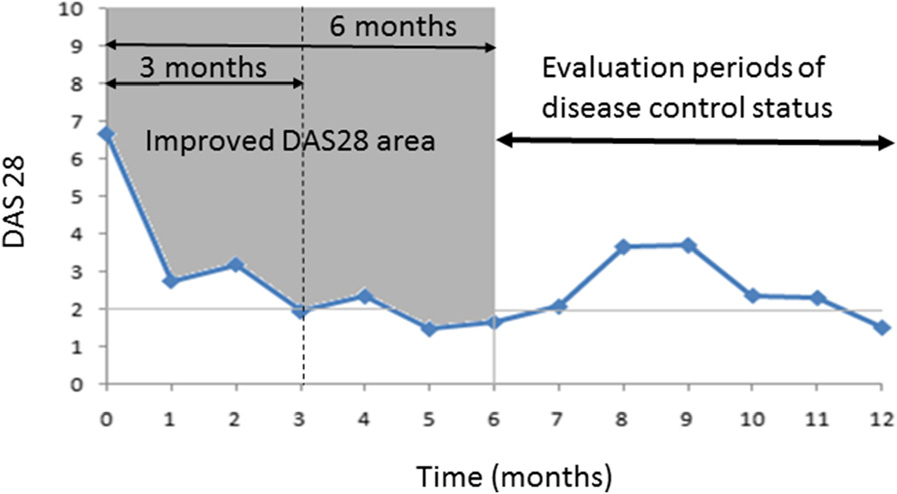
Figure Relationship between improved DAS28 area and evaluation periods of disease control status after the start of MTX administration. DAS28: Disease Activity Score-28 joints

Disease control was evaluated based on the value of DAS28-CRP for 6–12 months after the commencement of MTX administration (Fig.1). Good control was defined as when a patient received a fixed dose of MTX for 6–12 months after the start of administration (i.e., no change in the MTX dose) and DAS28 scores of >2 were recorded less than once per month on a day when a physician was visited. All other cases were defined as poor disease control.

The cumulative dose of MTX at 3 and 6 months after the start of MTX administration and the area under the curve of the DAS28 score (DAS28-AUC) were calculated using the trapezoidal rule for each patient. To adjust for the difference in the MTX treatment response due to the variation in patients’ DAS28 scores at the commencement of MTX administration, we assumed that a DAS28 score of 10 was maintained for 3 and 6 months, and in each case the total value of the DAS28 area was 30 during 0–3 months and 60 during 0–6 months, respectively. The improved DAS28 area was calculated by deducting each actual DAS28 area from the DAS28-AUC (30 in 3 months, 60 in 6 months). The value for the improved DAS28 area was divided by the cumulative dose of MTX, that is, we assumed that the improved DAS28 area per milligram of MTX was the index R of treatment response. The results in the good response group were then compared with those in the poor response group. That is, *R* = improved DAS28 area after the commencement of MTX administration during a 3-month or 6-month period/cumulative dose of MTX during the 3-month or 6-month period.

The evaluation periods for the index R of treatment response were 0–3 months and 0–6 months after the commencement of MTX administration.

#### Pharmacodynamic factors

As SNPs related to the MTX treatment response, RFC1 80G> A, FPGS 1994G> A, GGH –401C> T, methylenetetrahydrofolate reductase (MTHFR) 1298A > C, and thymidylate synthase (TYMS) 3′-UTR (+6/–6) were evaluated in all patients. The index R of treatment response was compared between the RFC1 80 G/G genotype and A allele, FPGS 1994G allele and A/A genotype, GGH −401 C/C genotype and T allele, MTHFR 1298A allele and C/C genotype, and TYMS 3′-UTR −6/–6 genotype and +6 allele.

#### Genotyping method

##### DNA extraction

For genetic analysis, 5 ml of peripheral blood was collected from each patient using the standard venipuncture technique in tubes containing ethylenediaminetetraacetic disodium salt and stored at −20 °C until DNA extraction. DNA was extracted using the Wizard Genomic DNA Purification Kit (Promega Corporation, Madison, WI, USA). DNA extraction procedures were performed following the manufacturer’s instructions. Total genomic DNA was quantified and its purity and integrity were analyzed using the GE Healthcare GeneQuan 1300 Spectrophotometer (Fisher Scientific, Pittsburgh, PA, USA).

##### Genotyping of alleles of the RFC1 80G> A, RFC1 –43 T > C, FPGS 1994G> A, GGH 401C> T, MTHFR 1298A> C, and TYMS 3′-UTR (−6/+6)

Genotyping of alleles of RFC1 80G > A (rs1051266), RFC1 –43 T > C (rs1131596), FPGS 1994G > A (rs10106), GGH 401C > T (rs3758149), MTHFR 1298A > C (rs1801131), and TYMS 3′-UTR (−6/+6) (rs16430) was performed using the TaqMan SNP Genotyping Assay from Applied Biosystems (Foster City, CA, USA) with fluorogenic binding probes. PCR amplification with the real-time PCR method was performed in 25 μL of reaction mixture including genomic DNA 20 ng (60 ng for FPGS 1994G > A), 0.63 μL of 40 × TaqMan SNP Genotyping Assay Mix (0.32 μL in RFC1 80G > A), and TaqMan Universal PCR Master Mix 12.5 μL. The reaction conditions of PCR were as follows: initial denaturation for 10 min at 95 °C; 40 cycles at 92 °C/15 s; and annealing and extension for 1 min at 60 °C (55 °C for FPGS 1994G > A). The PCR system used was the StepOnePlus real time PCR system (Applied Biosystems).

### Statistical analysis

Comparisons between cumulative doses of MTX, improved DAS28 areas, index R, and disease control status were performed using the Mann-Whitney *U*-test. For comparisons between index R for 0–6 months after the start of MTX administration and RFC1, FPGS, GGH, MTHFR, and TYMS genotypes, the Mann-Whitney *U*-test was performed. p Values of less than 0.05 were considered to represent statistically significant differences. All analyses were conducted using PASW Statistics 18 (SPSS Inc., Chicago, IL, USA).

## Results

### Study participants

A total of 21 RA patients (3 men, 18 women) were included in this study. Patient characteristics at the start of MTX administration are shown in Table 1. All patients were taking folic acid, but there was no concomitant use of DMARDs. Seven of 21 patients were judged as showing a good response, and the remaining 14 patients as showing a poor response.

**Table 1:** Patient clinical characteristics at the start of MTX administration

No. of patients	21
Women, %	85.7
Age (years)	57 (33–69)
DAS28-CRP	4.00 (2.31–6.66)
CRP (mg/dL)	1.26 (0.07–11.53)
ESR (mm/h)	30 (11–95)
RF (IU/mL)	85 (3–260)
Scr (mg/dL)	0.60 (0.45–1.02)
Duration of RA (months)	28 (3–237)
DMARD adminstration history (%)	73.7

Value at the start of MTX admistration: median (minimum–maximum)

*MTX* methotrexate, *DAS28-CRP* Disease Activity Score-28 joints–C-reactive protein, *ESR* erythrocyte sedimentation rate, *RF* rheumatoid factor, *Scr* serum creatinine, *RA* rheumatoid arthritis, *DMARD* disease-modifying antirheumatic drug

### Comparisons between cumulative dose of MTX, improved DAS28 area, index R, and disease control status

Table 2 shows comparisons between disease control status for 6–12 months and cumulative dose of MTX, improved DAS28 area, and index R for 0–3 months and 0–6 months after the commencement of MTX administration between the good control and poor control groups. For 0–3 months after starting MTX administration, the cumulative dose of MTX (median [25–75th percentile]) was 96.0 (94.0–116.0) mg in the good control group (*n* = 7) and 118.0 (100.0–124.0) mg in the poor control group (*n* = 14). The difference between the 2 groups was not statistically significant (*p* = 0.322). The improved DAS28 area was 25.4 (24.5–26.1) in the good control group (*n* = 7) and 23.4 (22.6–24.5) in the poor control group (*n* = 14). It was therefore significantly greater in the good control group (*p* = 0.004). Index R was 0.25 (0.22–0.27) in the good control group and 0.20 (0.19–0.24) in the poor control group. A tendency for a greater index R value was thus observed in the good control group (*p* = 0.079).

**Table 2:** Comparisons between disease control status during 6–12 months and cumulative dose of MTX, improved DAS28 area, and index R for 0–3 and 0–6 months after the start of MTX administration between the good and poor control groups

	Disease control status during 6–12 months		*p*-value
	Good (*n* = 7)	Poor (*n* = 14)	
0–3 months after the start of MTX administration			
Cumulative dose of MTX (mg)	96.0 (94.0–116.0)	118.0 (100.0–124.0)	0.322
Improved DAS28 area	25.4 (24.5–26.1)	23.4 (22.6–24.5)	0.004
Index R (1/mg)	0.25 (0.22–0.27)	0.20 (0.19–0.24)	0.079
0–6 months after the start of MTX administration			
Cumulative dose of MTX (mg)	192.0 (166.0–212.0)	214.0 (196.0–220.0)	0.287
Improved DAS28 area	51.0 (49.7–52.2)	47.6 (45.1–48.4)	0.001
Index R (1/mg)	0.26 (0.24–0.30)	0.22 (0.21–0.24)	0.025

median (25–75th percentile)

*MTX* methotrexate, *DAS28* Disease Activity Score-28 joints

For 0–6 months after starting MTX administration, the cumulative dose of MTX (median [25–75th percentile]) was 192.0 (166.0–212.0) mg in the good control group (*n* = 7) and 214.0 (196.0–220.0) mg in the poor control group (*n* = 14) (*p* = 0.287). The improved DAS28 area was 51.0 (49.7–52.2) in the good (*n* = 7) and 47.6 (45.1–48.4) in the poor control group (*n* = 14), meaning that the improvement was significantly greater in the former (*p* = 0.001). Index R was 0.26 (0.24–0.30) and 0.22 (0.21–0.24) in the good and poor control group, respectively (*p* = 0.025).

### Comparisons between index R for 0–6 months and RFC1, FPGS1994, GGH452, MTHFR1298, and TYMS 3′-UTR genotypes

Table 3 shows comparisons between index R for 0–6 months after the commencement of MTX administration and RFC1, FPGS1994, GGH452, MTHFR1298, and TYMS 3′-UTR genotypes. There were no statistically significant differences among them, although a slight tendency for a correlation between G/G genotypes and A allele genotypes in RFC1 80 genotypes was observed (*p* = 0.112).

**Table 3:** Comparisons between index R for 0–6 months after the start of MTX administration and RFC1, FPGS1994, GGH452, MTHFR1298, and TYMS 3'-UTR genotypes

Genotype		*n*	Index R	*p*-value
RFC1 80	G/G	6	0.22 (0.21–0.23)	0.112
	A allele	15	0.24 (0.22–0.30)	
FPGS 1994	G allele	17	0.23 (0.21–0.29)	0.517
	A/A	4	0.24 (0.23–0.28)	
GGH 452	C/C	11	0.23 (0.22–0.29)	0.973
	T allele	10	0.23 (0.22–0.26)	
MTHFR 1298	A allele	19	0.23 (0.22–0.28)	0.267
	C/C	2	0.27 (0.23–0.31)	
TYMS 3'-UTR	−6/–6	8	0.23 (0.22–0.23)	0.301
	+6 allele	13	0.25 (0.22–0.30)	

median (25–75th percentile)

*RFC1* reduced folate carrier 1, *FPGS* folypolyglutamate synthetase, *GGH* gammaglutamyl hydrolase enzyme, *MTHFR* methylenetetrahydrofolate reductase, *TYMS* thymidylate synthase

## Discussion

As there are large interindividual differences in the response to MTX treatment, we performed a preliminary study on predicting better MTX efficacy in Japanese patients with RA by investigating the relationship between treatment response and disease control status, along with genetic polymorphisms for MTX-related transporters/enzymes.

The good control group during 0–3 months and 0–6 months after the start of MTX administration showed a significantly greater improved DAS28 area than the poor control group (*p* = 0.004 for 0–3 months, 0.001 for 0–6 months). This result suggests that decreasing the DAS28 score aggressively during the early period of MTX administration contributes to better disease control and subsequent prognosis. Although the therapeutic effect of MTX is thought to depend on the dose, the cumulative MTX dose for 0–3 months and 0–6 months did not differ significantly difference between the good and poor control groups (*p* = 0.322 and 0.287, respectively). Therefore, individual differences in treatment response are related to the difference in the improved DAS28 area. Index R for 0–6 months was significantly greater in the good control group than in the poor control group (*p* = 0.025), and there was a nonsignificant tendency for index R to be greater in the good control group for 0–3 months (*p* = 0.079). Therefore, it appears necessary to tailor the optimal MTX dosage regimen to each patient because the treatment response differs among individuals.

Several groups have reported the factors predicting the response to MTX treatment, which focused on drug disposition including SNPs in genes coding for folate pathway enzymes and MTX transport into and out of cells in patients with RA in relation to MTX efficacy and toxicity [25–32]. Recently, for example, Kung et al. have reported that RFC1 80G > A was associated with MTX efficacy, but not toxicity [25]. Moya et al. reported that two FPGS SNPs (rs10987742 and rs10106) were associated with treatment response and that ABCB1 SNPs (rs868755, rs10280623, rs 1858923) were associated with toxicity [26]. Ghodke-Puranik et al. found that SNPs in MTHFR and RFC1 were associated with MTX efficacy and SNPs in GGH, SHMT1, and TS with MTX-related adverse events [27]. The results of Świerkot et al. showed that MTHFR 677CC, GGH 401TT, and CT genotypes were associated with a reduction in the number of MTX-related adverse events [28]. Our results of comparisons between index R for 0–6 months after starting MTX administration and RFC1 80 G > A, FPGS 1994 G > A, GGH −401 C > T, MTHFR 1298 A > C, and TYMS 3′-UTR (−6/+6) did not show statistically significant differences or clear correlations among them. For this reason, we cannot rule out a type II error in our study, i.e., that the sample was too small to detect significant differences, because RFC1 80G > A genotypes may be slightly correlated with index R (*p* = 0.112), although no SNPs were. The lack of a significant difference in the cumulative dose of MTX between the good and poor control groups, but a significant difference in the improved DAS28 area and/or index R between them, suggests that these SNPs may contribute to better disease control status at 6–12 months. It is necessary to study this in a larger patient population including single-gene polymorphisms and other factors associated with treatment response.

This study had several limitations. First, the disease control evaluation period was 6–12 months after the commencement of MTX administration. RA is a chronic disease, however, and it has been reported that patients with RA continue receiving MTX significantly longer than other DMARDs, with 60–70 % of them administered MTX for as long as 5.0–7.5 years [34]. This needs to be observed over longer-term follow-up. Second, we defined the disease control status as “good” when the MTX dose was fixed and patients had clinically stable, well-controlled symptoms, i.e., no dose change was necessary during the study period, and/or the number of days patients had a DAS28 score >2 was fewer than once for 6–12 months after the start of MTX administration. These criteria for disease control status would be difficult to apply in clinical practice. In previous studies using the DAS as an index, improvement from baseline to 3 or 6 months was observed [30, 35–38]. In addition, in one study responders were defined as patients with a DAS28 ≤ 3.2 and nonresponders as those with a DAS28 > 2.8 [35]; in another studies, responders were patients with a DAS <2.4 (good clinical response) and nonresponders were those with a DAS >2.4 based on the European League Against Rheumatism (EULAR) response criteria [30, 36–38]. Our definition of maintaining a DAS28-CRP ≤2.0 continuously for 6–12 months is stricter. Therefore, it should be confirmed whether this would be appropriate in clinical practice. Furthermore, the present results should be compared with the results of EULAR improvement criteria, the change in DAS28 from baseline with MTX administration, and with ACR20, 50, and 70, along with other treatment response criteria used in previous studies to determine whether they are equivalent. Third, we did not evaluate whether disease control could be achieved with lower doses of MTX in the good control group, or whether it could be achieved with higher doses in the poor control group, because this was a pragmatic observational study. Further study will be needed to confirm this possibility. Fourth, we excluded patients with adverse drug reactions to MTX, because we focused only on a method for predicting the efficacy of MTX treatment of RA in this study. The clinical application of this “aggressive” RA treatment will need to consider the balance between MTX efficacy and safety. Further study is therefore needed to investigate the dose of MTX in aggressive RA treatment including patients with adverse drug reactions to MTX and to determine which patients should not receive aggressive treatment since they are likely to have adverse reactions. Fifth, the individual differences in treatment response are dependent on susceptibility to treatment or MTX pharmacokinetics, and there are some reports that MTX polyglutamation concentration in red blood cells may be correlated with efficacy in adult patients with RA [29, 39–41], although that concentration was not measured in this study and the correlation was not investigated. The relationship between treatment effect and MTX polyglutamation concentration in red blood cells should therefore be clarified. Finally, the sample size was too small and sensitivity analysis to confirm index R was not performed in this study because it was a retrospective cohort design, and it will therefore be necessary to undertake a prospective study in a larger patient population in the future, including sensitivity analysis and the SNPs related to MTX treatment response studied here.

## Conclusion

This study suggested that aggressive RA treatment with MTX administration from the early period is needed to obtain a good response after 6 months, although SNPs for MTX-related transporters/enzymes to predict a better treatment response to MTX were not identified. Further study in a larger sample is planned.
